# The prevalence of soil transmitted helminth infections in minority indigenous populations of South-East Asia and the Western Pacific Region: A systematic review and meta-analysis

**DOI:** 10.1371/journal.pntd.0009890

**Published:** 2021-11-10

**Authors:** Beth Gilmour, Kefyalew Addis Alene, Archie C. A. Clements

**Affiliations:** 1 Faculty of Health Sciences, Curtin University, Western Australia, Australia; 2 Telethon Kids Institute, Nedlands, WA, Australia; Hospital General, MEXICO

## Abstract

**Introduction:**

Soil transmitted helminth (STH) infections cause one of the most prevalent diseases in man. STHs disproportionately impact socio-economically disadvantaged communities including minority indigenous populations. This systematic review aimed to quantify the prevalence of STH infection within minority indigenous populations of the South-East Asia and Western Pacific Regions.

**Methods:**

The systematic review was conducted in accordance with The Preferred Reporting Items for Systematic Reviews and Meta-Analyses (PRISMA) guidelines following a published protocol. A random effects meta-analysis was used to estimate the pooled prevalence of STH infection, and meta-regression analysis was used to quantify associations with study characteristics. Where comparative data were available, sub-group analysis was conducted to evaluate the risk of STH infection in minority indigenous people relative to other population groups. The heterogeneity between studies was evaluated visually using Forest plots and was assessed quantitatively by the index of heterogeneity (I^2^) and Cochran Q-statistics.

**Results:**

From 1,366 unique studies that were identified, 81 were included in the final analysis. The pooled prevalence of infection within minority indigenous populations was 61.4% (95% CI 50.8, 71.4) for overall STH infection; 32.3% (95% CI 25.7, 39.3) for *Ascaris*.*lumbricoides*; 43.6% (95% CI 32.6, 54.8) for *Trichuris*.*trichiura*; 19.9% (95% CI 15.7, 24.5) for hookworm and 6.3% (95% CI 3.2, 10.2) for *Strongyloides*.*stercoralis*. A significant increase in *T*. *trichiura* prevalence was observed over time. The stratified analysis showed that the prevalence of infection for STH overall and for each STH species were not significantly different in minority indigenous participants compared to other populations groups.

**Conclusion:**

The prevalence of STH infection is high within minority indigenous populations across countries at very different levels of socio-economic development. The increasing prevalence of *T*. *trichiura* calls for the implementation of more effective therapies and control strategies.

## Author summary

Neglected Tropical Diseases (NTD) are caused by a range of infectious pathogens and have their greatest impact on poor and vulnerable populations. One such population group, is indigenous people, who are disproportionately impacted by poverty and social disadvantage. Among the World Health Organization (WHO) list of 20 NTD, soil transmitted helminth (STH; *Ascaris lumbricoides*, *Trichuris trichiura* and hookworm) infections are the most prevalent and burdensome. This systematic review and meta-analysis evaluated the prevalence of STH infection in minority indigenous populations of the South-East Asia (SEAR) and Western Pacific Regions (WPR). The results showed a high prevalence of infection for all STH species in minority indigenous populations of both developing and highly industrialized nations. Of concern was the increasing prevalence of *T*. *trichiura* infection over time, which calls for the identification and implementation of more effective therapies and control strategies. Where comparative data were available, the review showed infection prevalence of all STH species not to be significantly different in minority indigenous people compared to those of other population groups. To help break the health burden and poverty cycle created by these infections, accurate, relevant data will be required to inform effective and appropriate interventions.

## Introduction

Soil transmitted helminthiasis is a Neglected Tropical Disease (NTD)[1] estimated to impact 1.5 billion people,[2] a figure which equates to 19% of the world’s population. The four species of gastro-intestinal nematode commonly included in soil transmitted helminths (STH) are *Ascaris lumbricoides* (roundworms), *Trichuris trichiura* (whipworms), *Necator americanus* and *Ancylostoma duodenale* (hookworms). *Ancylostoma ceylanicum* is also an increasingly recognized hookworm species of public health importance. These parasites prevail in the tropics and subtropics and have their greatest impact on populations affected by poverty and disadvantage.[2–5]

The impact STH infection creates a significant global health burden. In 2016, the WHO estimated a loss of 3.4 million disability adjusted life-years (DALY) worldwide, of which 42% was attributed to *A*. *lumbricoides*, 10% to *T*. *trichiura* and 48% to hookworm infection.[6] A significant proportion of the total disease burden is attributed to Years Lost due to Disability (YLD) which is estimated at 2.9 million.[6]

The quantum of the YLD estimate is reflective of the chronic and debilitating morbidity associated with STH infections. The symptoms of morbidity are often difficult to quantify due to the effects of poverty, malnutrition and co-infection, which are common amongst those worst affected.[7] However, a number of morbidities have been well documented and include impaired growth and physical development, intestinal obstruction, anaemia, vitamin A deficiency, and poor intellectual and cognitive development.[4,8]

Although not as well represented in the literature, *Strongyloides stercoralis* is another pathogenic STH of significance to human health. While the prevalence of *Strongyloidiasis* is difficult to quantify due to many cases being asymptomatic and traditional diagnostic methods lacking sensitivity,[9] global estimates project between 100 and 370 million infections.[9,10] *S*. *stercoralis* is differentiated from other STH species by an auto-infective capability within the lifecycle[11] and by its prevalence in both tropical and temperate climates.[12] Statistics for *S*. *stercoralis* are not included within DALY figures and although hyper-infection syndrome for this parasite is rare, it is often fatal in immunocompromised patients among whom mortality rates of 86% are reported.[13]

The successful control of STH infections will be dependent upon a multi-faceted approach. Economic development is proven to be a significant factor in eradicating STH infections[14] and it is acknowledged that WASH (water, sanitation and hygiene) and education initiatives are fundamental to reducing disease transmission.[15] These approaches are combined with the primary focus of the WHO endorsed control strategy for *A*. *lumbricoides*, *T*. *trichiura* and hookworm infection which is the periodic administration of anthelmintic drugs to at-risk populations living in endemic areas.[16] Although well-developed treatment strategies have been developed for *A*. *lumbricoides*, *T*. *trichiura* and hookworm,[15] systematic action plans to address *Strongyloidiasis* are lacking.[10] There is a fundamental lack of epidemiological data for *Strongyloides* infection, a knowledge gap not limited to developing regions as evidenced by the call for its inclusion on the Australian Notifiable Disease List.[17]

Although significant reductions in STH prevalence have been achieved over recent times,[18] infections continue to impose a significant global health burden and impact those most vulnerable within society. One population group that has been shown to be disproportionately affected by poverty and social disadvantage is indigenous people.[19]

Although there are published studies on the impact of STH infections within discrete ethnic groups, there is nothing in the literature that quantifies STH infection risk in minority indigenous people as a collective. If the goals of the 2030 Agenda for Sustainable Development are to be achieved, the burden of disease amongst vulnerable populations needs to be evaluated to inform effective interventions.

This systematic review aimed to quantify the prevalence of STH infection amongst minority indigenous populations of the SEAR and WPR. These regions were chosen as WHO data attributes a high proportion of DALYs to be lost as a result of STH infection within these areas.[6]

The SEAR and WPR also include a significant representation of indigenous populations[20] whilst providing an opportunity to compare the prevalence of STH infection across countries of differing socio-economic strata.

## Methods

### Search strategy

The systematic review was undertaken in accordance with the Preferred Reporting Items for Systematic Reviews and Meta-Analyses (PRISMA) guidelines[21] (S1 PRISMA checklist). Specifics on the search criteria and details of the study selection criteria are available in a published protocol.[22]

In summary, four biomedical databases: Scopus, Web of Science, Medline (Ovid) and EMBASE (Ovid), were systematically searched using the criteria detailed in S1 Table, without restriction on the year of publication. In addition to the biomedical database search, reference lists from included publications were hand searched.

### Study screening and selection criteria

All studies identified from the systematic search were imported into Endnote X9 (Clarivate Analytics) where duplicates were deleted. Following removal of the duplicates, studies were uploaded to Rayyan Qatar Computing Research Institute (QCRI) [23] and titles and abstracts were independently assessed by two authors (BG and KAA). The full text articles of shortlisted abstracts were independently screened by the same two authors against the inclusion and exclusion criteria.

Any discrepancies relating to the shortlisting of publications were discussed and where consensus could not be achieved, advice was sought from the third author (ACAC). Where further clarification was required, this was requested from the corresponding author of the relevant publication.

### Inclusion criteria

Studies were included if they were representative cross-sectional surveys relating to human infection and provided sufficient data to facilitate the calculation of STH prevalence. Studies were required to include minority indigenous population participants within the SEAR or WPR.

In accordance with the protocol,[22] minority indigenous populations were defined when each of the following criteria were met:

■ Descendants of the original or earliest known inhabitants of an area; people who have historical continuity with pre-invasion and pre-colonial societies.
■ Distinct societies with languages, culture, customs, and social and political frameworks that vary significantly from those of the dominant population.
■ Groups of people with strong cultural ties and dependence upon the environment and its resources for their survival.
■ People self-identifying as indigenous.
■ Groups who face relative disadvantage or discrimination in multiple areas of social existence- success, education, healthcare, employment.
■ Numerically non-dominant groups in a country or area.

The WHO Global Burden of Disease (GBD) regional classification system [24] was used to define the countries located within the SEAR and WPR.

### Exclusion criteria

Studies were excluded if they were not full text articles and did not publish in English. Publications were excluded if less than 90% of the participants (or, for the comparative studies, the minority indigenous category) met the minority indigenous population criteria. Data from case series with less than 10 participants and case studies; systematic and literature reviews; conference poster or abstracts and scientific correspondence e.g., letters to the editor, were excluded. Singapore was excluded from the search as it does not have any minority indigenous people according to the definitions used by this review.

### Outcomes

The primary outcome of the study was prevalence of STH infection amongst minority indigenous populations of the SEAR and WPRs. Prevalence included STH infection overall and according to species: *A*.*lumbricoides*, *T*.*trichiura*, *S*.*stercoralis* and Hookworm species collectively.

### Data extraction and quality assessment

Data were extracted from included studies using Microsoft Excel version 2016 (Microsoft, Redmond, Washington, USA) by BG and independently validated by KAA. Following pilot testing and refinement, a data extraction spreadsheet was used to record the following information: first author and year of publication; year and country in which the study was undertaken; study population classification (minority indigenous or other); species of infectious agent; diagnostic method; sex of study participants; size (n) of the study population and number of disease positive participants. Although the protocol [22] also intended to extract and analyze data by age, this was not undertaken due to the large variation in age classifications across publications.

Where studies evaluated the impact of intervention regimes, only pre-intervention baseline data were extracted. When surveys undertook a comparison of disease prevalence across minority indigenous and other population groups, data were extracted for both to facilitate a comparison.

A modified version of the Newcastle-Ottawa Quality Assessment (QA) Scale[25] was utilized to assess the quality of the studies analysed, the scores for which are detailed in S2 Table. The QA tool has scores ranging from 0 to 9, in accordance with the protocol,[22] scores between 1 and 4 were defined as low quality, scores between 5 and 7 were defined as medium quality, and scores between 8 and 9 defined as high quality.

### Study variables

The mortality strata for each country of study was attributed according to the WHO definitions[26], and was evaluated as a study variable. The other study variables used for the sub-group analysis included: WHO region, country of study, year of data collection, study location (community/school), number of samples analysed (singular/multiple), diagnostic method, number of helminth infections, study participant sex, helminth species (for hookworm) and QA grade.

### Data analysis

For the studies that identified overall STH infection, and for data extracted by species (*A*.*lumbricoides*, *T*.*trichiura*, hookworm and *S*.*stercoralis*), a random-effects meta-analysis was used to estimate the pooled prevalence of infection. The meta-analysis was undertaken using the Freeman-Tukey double arcsine transformation to address confidence limits outside the 0 to 1 range and variance instability.[27] This was implemented in Stata using the *metaprop* command.[28]

The heterogeneity between studies in minority indigenous populations was assessed using Cochran’s Q test and was quantitatively evaluated with the index of heterogeneity squared (I^2^) statistic with 95% CI.[29] Heterogeneity between studies was classified low, moderate and high when I^2^ values were below 25%, between 25% and 75% and above 75%, respectively.[29]

In an attempt to account for the high heterogeneity that was identified, meta-regression was undertaken using the study characteristics as covariates. The meta- regression was conducted using the robust variance estimation (RVE) method to manage non-independent effect sizes without knowledge of the within-study covariance structure.[30]

Where comparative data were available, sub-group analysis was conducted to evaluate the risk of helminth infection in minority indigenous communities relative to other population groups. Where differences in infection prevalence were identified across study variables, or between population groups, bivariate meta-regression was used to evaluate their significance (*p*-value <0.05) when three or more data sets were available for each comparison.

Funnel plots were utilized to evaluate potential publication bias and asymmetry was assessed using Egger’s method with a *p*-value <0.05 denoting significant bias.[31] Analysis was conducted using Stata/MP version 16.1 (StataCorp, College Station, TX).

## Results

**Fig 1:**
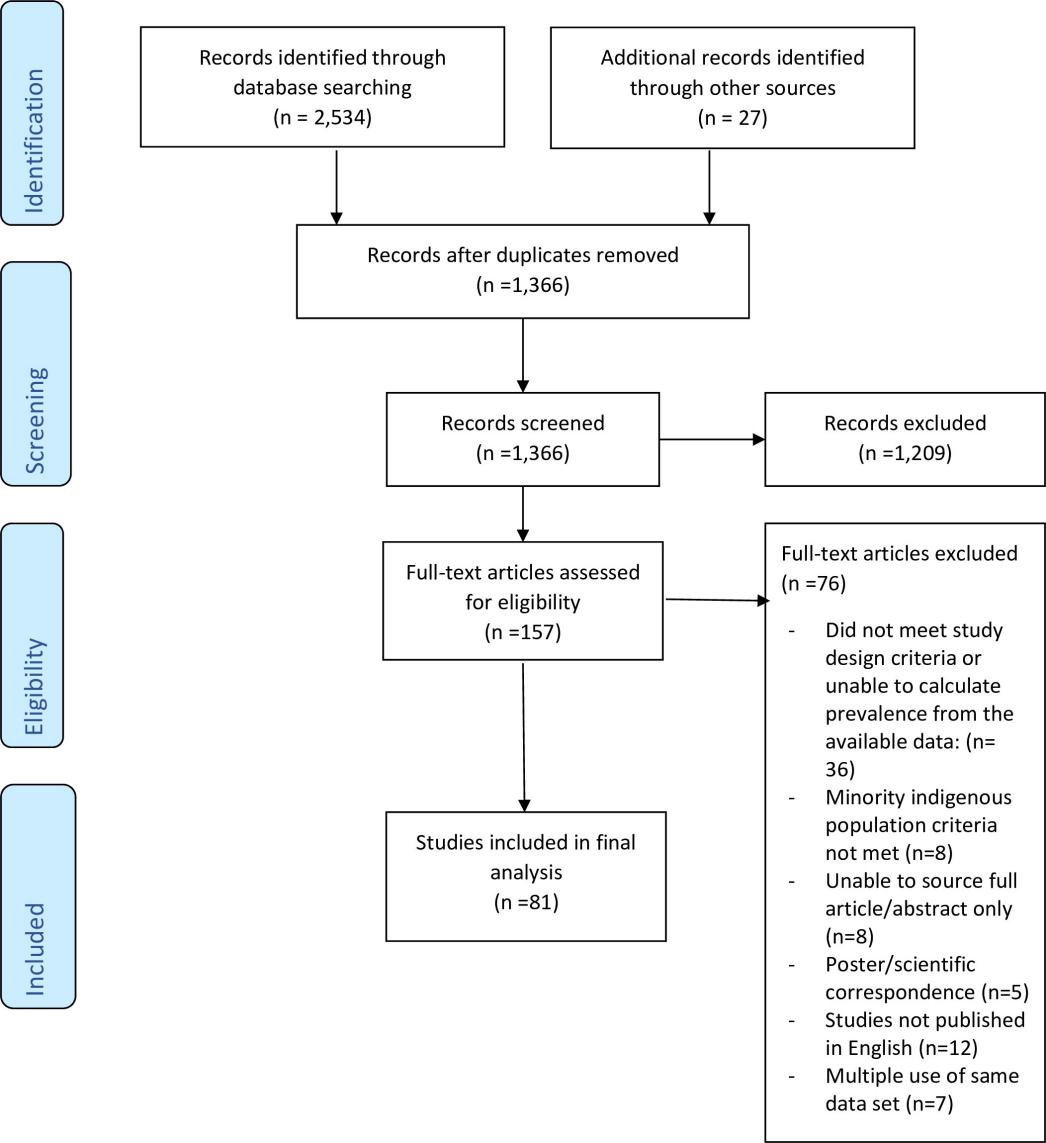
Summary of PRISMA systematic review publication selection process.

**Fig 2:**
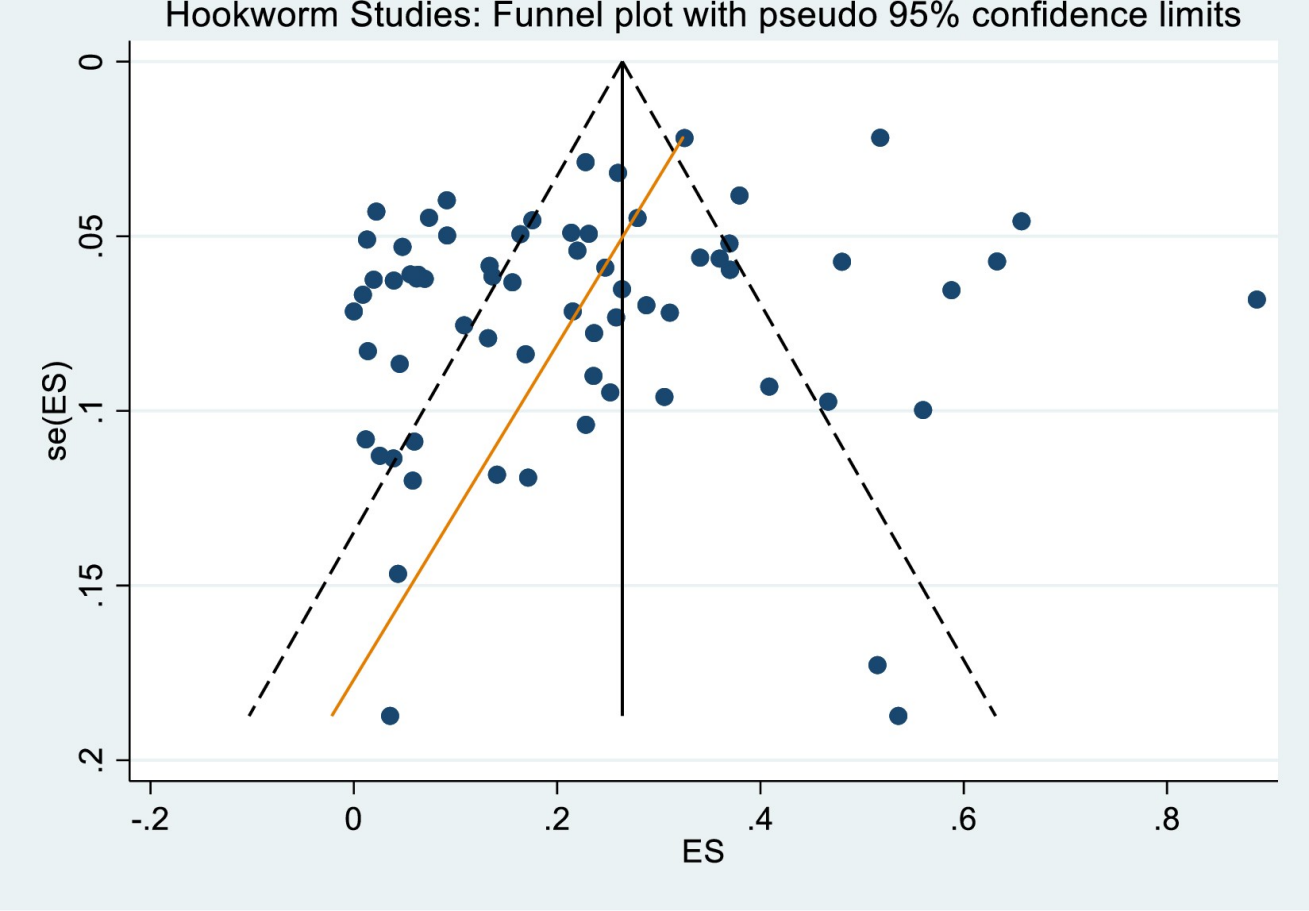
Funnel plot of hookworm* studies with pseudo 95% confidence intervals. *The hookworm data set was used to assess publication bias as this contains the largest number of studies (68 of the 81). Egger’s test produced a bias coefficient of -2.09 (95% CI -3.90, -0.28) p-value 0.025 indicating publication bias.

The search identified 1,366 unique studies from which 157 were shortlisted following title and abstract screening. Following the full text review, 81 studies were included in the final analysis (Fig 1); the characteristics of the studies are provided in Table 1. Publication bias of the included studies was evidenced by the asymmetrical shape of the funnel plot (Fig 2) and a *p* value = 0.025 calculated with Egger’s regression test.

**Table 1:** Summary of STH studies within minority indigenous populations in South-East Asia and the Western Pacific Region.

Study ID	First Author Year of Publication	Year of Data Collection ^Δ^	WHO Region	WHO Mortality Strata	Country	STH species	Study Population size (n)	Number Positive ^	% Male	Median Age or *Mean Age
1	Adli, 2019	<2019	WPR	B	Malaysia	Hookworm	71	10		
2	Adli, 2020	2017	WPR	B	Malaysia	*A*.*lumbricoides/T*.*trichiura*/Hookworm	92	70		
3	Ahmad, 2013	<2013	WPR	B	Malaysia	*S*.*stercoralis*	54	3		
4	Ahmed, 2011	2010	WPR	B	Malaysia	*A*.*lumbricoides/T*.*trichiura*/Hookworm	254	238	48.8	9.5
5	Al-Delaimy, 2014A	<2014	WPR	B	Malaysia	*Trichuriasis/Ascariasis*/Hookworm	317	315	48.9	9
6	Al-Delaimy, 2014B	2012	WPR	B	Malaysia	*A*.*lumbricoides/T*.*trichiura*/Hookworm	498	490	50.6	9
7	Al-Mekhlafi, 2005	<2005	WPR	B	Malaysia	*Trichuriasis/Ascariasis*/Hookworm	368		48.7	* 7.1
8	Al-Mekhlafi, 2006	<2006	WPR	B	Malaysia	*A*.*lumbricoides/T*.*trichiura*/Hookworm	281	281	50.9	
9	Al-Mekhlafi, 2007	2006	WPR	B	Malaysia	*A*.*lumbricoides/T*.*trichiura*/Hookworm	292	288	49.7	9.6
10	Al-Mekhlafi, 2019	2017	WPR	B	Malaysia	*S*.*stercoralis*	1142	180	49.4	*10.19
11	Anuar, 2014	<2014	WPR	B	Malaysia	*A*.*lumbricoides/T*.*trichiura*/Hookworm	500		43.8	
12	Ash, 2017	2013	WPR	B	Laos	*A*.*lumbricoides/T*.*trichiura*	100	90		
13	Bangs, 1996	1990	SEAR	B	Indonesia	*A*.*lumbricoides/T*.*trichiura*/Hookworm/*S*.*stercoralis*	478			
14	Belizario, 2011	2009	WPR	B	Philippines	*A*.*lumbricoides/T*.*trichiura*/Hookworm	264	103	43.9	*10.08
15	Brandon-Mong, 2017	2013–2014	WPR	B	Malaysia	*A*.*lumbricoides/T*.*trichiura*/Hookworm	235	192	50.2	26
16	Chakma, 2000	<2000	SEAR	D	India	*A*.*lumbricoides*/hookworm	409			
17	Chin, 2016	2014	WPR	B	Malaysia	*A*.*lumbricoides/T*.*trichiura/A*.*ceylancum/A*.*americanus*	186	114	42.5	26
18	Choubisa, 1992	<1992	SEAR	D	India	*A*.*lumbricoides/N*.*americanus/A*.*doudenale/T*.*trichiura*	250			
19	Choubisa, 2012	2010–2011	SEAR	D	India	*A*.*lumbricoides/A*.*duodenale/S*.*stercoralis/T*.*trichiura*	224		51.3	
20	Damon, 1974	1966 + 1968	WPR	B	Solomon Isl	*A*.*lumbricoides/T*.*trichiura*/Hookworm	105			
21	DeGuia, 2019	<2019	WPR	B	Philippines	*Ascaris/Trichuris*/Hookworm	223	159		
22	Elyana, 2016	2014–2015	WPR	B	Malaysia	*A*.*lumbricoides/T*.*trichiura*/Hookworm	165		53.3	
23	Farook, 2002	2001	SEAR	D	India	Roundworm/Hookworm/*Strongyloides*/Whipworm	258	60	44.6	
24	Fryar, 1997	1996	WPR	A	Australia	*T*.*trichiura/Strongyloides*/Hookworm	28	9		
25	Geik, 2015	2014	WPR	B	Malaysia	*A*.*lumbricoides/T*.*trichiura*/Hookworm	256	161	51.2	* 3.2
26	Ghani, 2013	<2012	WPR	B	Malaysia	*A*.*lumbricoides*	272	124	47.4	
27	Hall, 1994	<1994	SEAR	D	Bangladesh	*S*.*stercoralis*	656	89		
28	Hanapian, 2014	2005–2006	WPR	B	Malaysia	*A*.*lumbricoides/T*.*trichiura*/Hookworm	175	131	49.7	15.11
29	Hartini, 2013	<2013	WPR	B	Malaysia	*A*.*lumbricoides/T*.*trichiura*/Hookworm	111			
30	Holt, 2017	2010–2011	WPR	A	Australia	*T*.*trichiura*/Hookworm/*S*.*stercoralis*	85			3.7
31	Hung, 2016	2015	WPR	B	Vietnam	*A*.*lumbricoides/T*.*trichiura*/Hookworm	1206	301		
32	Kaliappan, 2013	2011–2012	SEAR	D	India	*A*.*lumbricoides/T*.*trichiura*/Hookworm	680	265		
33	Kalra, 1982	1979	SEAR	D	India	*A*.*lumbricoides/T*.*trichiura*/Hookworm	115			
34	Kearns, 2017	2010	WPR	A	Australia	*S*.*stercoralis*	818	185	49	21
35	Lee, 2014	2010–2012	WPR	B	Malaysia	*Ascaris spp/T*.*trichiura*/Hookworm	269	149		
36	Lili, 2000	1998	WPR	B	China	*Ascaris spp/Trichuris*/Hookworm	304	219		
37	Lyndem, 2002	1996–1999	SEAR	D	India	*N*.*americanus/Ascaris/Trichuris*	2087		51.6	
38	Meloni, 1993	1987–1991	WPR	A	Australia	*A*.*duodenale/T*.*trichiura/S*.*stercoralis*	385			
39	Miller, 2018	2004–2005	WPR	A	Australia	*S*.*stercoralis*	867	144	46	
40	Mohd-Shadaruddin, 2018	2014–2015	WPR	B	Malaysia	*A*.*lumbricoides/T*.*trichiura*/Hookworm	411	299	48.8	4
41	Muslim, 2019	2016–2017	WPR	B	Malaysia	*A*.*lumbricoides/T*.*trichiura*/Hookworm/*S*.*stercoralis*	416	358	50	10
42	Nasr, 2013	2011	WPR	B	Malaysia	*A*.*lumbricoides/T*.*trichiura*/Hookworm	484	378	51.4	7
43	Neo, 1987	<1987	WPR	B	Malaysia	*A*.*lumbricoides/T*.*trichiura*/Hookworm	142	92		
44	Ng, 2014	2011	WPR	B	Philippines	*A*.*lumbricoides/T*.*trichiura*/Hookworm	195	190	42	
45	Ngui, 2015	2009–2011	WPR	B	Malaysia	*A*.*lumbricoides/T*.*trichiura*/Hookworm	634	380	43.5	11
46	Ngui, 2016	<2016	WPR	B	Malaysia	*S*.*stercoralis*	236	26	53	44
47	Nithikathkul, 2003	2002	SEAR	B	Thailand	*A*.*lumbricoides/T*.*trichiura*/Hookworm/*S*.*stercoralis*	70		48.6	
48	Nithikathkul, 2007	2002	SEAR	B	Thailand	*T*.*trichiura*/Hookworm	133	15	45.9	
49	Nor Aini, 2007	2003–2004	WPR	B	Malaysia	*A*.*lumbricoides/T*.*trichiura*/Hookworm	281	281		
50	Norhayati, 1995	<1995	WPR	B	Malaysia	Hookworm	193	60	48.2	
51	Norhayati, 1997	<1997	WPR	B	Malaysia	*Ascaris spp/Trichuris*/Hookworm	123			
52	Norhayati, 1998	<1997	WPR	B	Malaysia	*Ascaris spp/Trichuris*/Hookworm	205		46.3	
53	Piangjai, 2003	1997–1998	SEAR	B	Thailand	*A*.*lumbricoides/T*.*trichiura*/Hookworm	403		48.9	
54	Prownebon, 2013	2008	SEAR	B	Thailand	*A*.*lumbricoides/T*.*trichiura*/Hookworm	145		48.3	
55	Rahmah, 1997	1996	WPR	B	Malaysia	*A*.*lumbricoides/T*.*trichiura*/Hookworm/*S*.*stercoralis*	84	67		
56	Rajeswari, 1994	<1994	WPR	B	Malaysia	*A*.*lumbricoides/T*.*trichiura*/Hookworm	78			
57	Rajoo, 2017	<2017	WPR	B	Malaysia	*A*.*lumbricoides/T*.*trichiura*/Hookworm	341	195	45.5	30
58	Ranjitkar, 2014	2011	SEAR	D	Nepal	STH	27	5		
59	Rao, 2002	2000–2001	SEAR	D	India	*Ascaris*/Hookworm	985			
60	Rao, 2006	1997	SEAR	D	India	*A*.*lumbricoides/T*.*trichiura*	40	40		
61	Reynoldson, 1997	1996	WPR	A	Australia	*A*.*duodenale/T*.*trichiura/S*.*stercoralis*	108			
62	Ribas, 2017	<2017	WPR	B	Laos	*A*.*lumbricoides/T*.*trichiura*/Hookworm/*S*.*stercoralis*	305	210		
63	Ritchie, 1954	1949	WPR	A	Japan	*A*.*lumbricoides/T*.*trichiura*/Hookworm	195			
64	Sagin, 2002	<2002	WPR	B	Malaysia	*A*.*lumbricoides/T*.*trichiura*/Hookworm	355			
65	Saksirisampant, 2004	2002–2003	SEAR	B	Thailand	*A*.*lumbricoides/T*.*trichiura*/Hookworm/*S*.*stercoralis*	542		40.6	
66	Shield, 2015	1994–1996	WPR	A	Australia	*T*.*trichiura/*Hookworm/*S*.*stercoralis*	314	276		
67	Singh, 1993	<1993	SEAR	D	India	*A*.*lumbricoides/T*.*trichiura*/Hookworm/*S*.*stercoralis*	28			
68	Sinniah, 2012	2011	WPR	B	Malaysia	*A*.*lumbricoides/T*.*trichiura*/Hookworm	77	36	31	
69	Sinniah, 2014	<2014	WPR	B	Malaysia	*A*.*lumbricoides/T*.*trichiura*/Hookworm	106			
70	Stafford, 1980	<1980	SEAR	B	Indonesia	*A*.*lumbricoides/T*.*trichiura*/Hookworm	287			
71	Steinmann, 2008	2006	WPR	B	China	*A*.*lumbricoides/T*.*trichiura*/Hookworm/*S*.*stercoralis*	215		47.4	* 29
72	Sugunan, 1996	<1996	SEAR	D	India	*A*.*lumbricoides/T*.*trichiura*/Hookworm	46			
73	Tienboon, 2007	<2007	SEAR	B	Thailand	*A*.*lumbricoides/T*.*trichiura*/Hookworm/*S*.*stercoralis*	158		52.5	
74	Verle, 2003	1999	WPR	B	Vietnam	*A*.*lumbricoides/T*.*trichiura*/Hookworm	2103			
75	Wong, 2016	<2016	WPR	B	Malaysia	*A*.*lumbricoides/T*.*trichiura*/Hookworm	33	32	58	
76	Yanola, 2018	2015–2016	SEAR	B	Thailand	*A*.*lumbricoides/T*.*trichiura*	375	33	37	
77	Yap, 2012	2011	WPR	B	China	*A*.*lumbricoides/T*.*trichiura*/Hookworm	69	59	42	11
78	Yoshida, 1968	1966	WPR	B	Taiwan	*Ascaris spp/Trichuris*/Hookworm	233			
79	Zulkifli, 1999A	<1999	WPR	B	Malaysia	*A*.*lumbricoides/T*.*trichiura*/Hookworm	268	127	49.6	
80	Zulkifli, 1999 B	<1999	WPR	B	Malaysia	*A*.*lumbricoides/T*.*trichiura*/Hookworm	259	145		
81	Zulkifli, 2000	<2000	WPR	B	Malaysia	*A*.*lumbricoides/T*.*trichiura*/Hookworm	123	86		

Notes

^ Where the number of participants positive for STH is not detailed, the study details data by species

^Δ^ Where the study does not detail the year of data collection, it is assumed < year of publication

### Prevalence of overall STH Infection

Out of the 81 studies, 49 enabled the overall prevalence of STH infection to be calculated. Details on the pooled prevalence of infection and bivariate meta-regression across the study covariates are detailed in Tables 2 and 3, respectively.

**Table 2:** Pooled prevalence of STH infections analysed by study covariates.

Categories	Pooled prevalence of STH^Δ^ Infection		
	Studies (n)	Pooled Prevalence (95% CI)	
**Population group**			
Minority indigenous populations	49	61.38 (50.82, 71.42)	
**Comparative Studies**			
Non-minority indigenous populations	5	37.46 (10.57, 69.45)	
Minority indigenous populations	5	41.93 (15.63, 70.94)	
	**Analysis on minority indigenous populations only**		
**WHO regions**			
SEAR	7	30.27 (15.62, 47.28)	
WPR	42	66.31 (55.24, 76.55)	
**WHO Mortality Strata**			
A	4	39.98 (10.89, 73.59)	
B	40	65.82 (54.36, 76.43)	
D	5	40.78 (20.33, 63.02)	
**Countries**			
Australia	4	39.98 (10.89, 73.59)	
Bangladesh	1	NA	
China	2	74.82 (70.25, 79.14)	
India	3	59.22 (27.71, 87.07)	
Laos	2	74.84 (70.48, 78.98)	
Malaysia	30	68.36 (55.38, 80.04)	
Nepal	1	NA	
Philippines	3	73.34 (33.34, 98.45)	
Thailand	2	9.37 (6.96, 12.09)	
Vietnam	1	NA	
**Year of data collection**			
1981–2000	11	61.59 (41.78, 79.60)	
2001–2020	38	61.30 (48.92, 73.00)	
**Study Location**			
Community	35	56.57 (45.39, 67.42)	
School	14	72.90 (48.59, 91.59)	
**Number of samples analysed**			
Singular	47	62.95 (52.10, 73.18)	
Multiple	2	25.42 (23.12, 27.79)	
**Diagnostic method** *			
Microscopy	44	65.97 (55.08, 76.08)	
PCR	2	46.72 (40.39, 53.10)	
Serology	3	16.78 (11.43, 22.92)	
**QA Grade**			
Low	5	60.24 (31.92, 85.37)	
Medium	40	59.24 (48.32, 69.72)	
High	4	81.81 (27.17, 100.00)	

Notes

^Δ^ STH prevalence: Overall prevalence is only available for 49 of the 81 studies, the balance of publications present data at species level. For the calculation of overall STH prevalence, 49 studies detailed the summary level of infection when multiple species were investigated, or the studies were based on a single helminth species.

*Diagnostic method: PCR and microscopy classified as PCR; ELISA classified as serology

**Table 3:** Bivariate meta-regression of STH infections analysed by study covariates.

Categories	Pooled prevalence of STH Infection		
	95% CI	*p* value	I^2^ ^α^ (%)
**Comparative Studies**			95.93
Non-Minority indigenous populations	1.00		99.26
Minority indigenous populations	1.03 (0.67, 1.59)	0.870	99.03
**Analysis on minority indigenous populations only**			
**WHO regions**			96.24
SEAR	1.00		99.41
WPR	1.39 (1.09, 1.77)	0.010	98.29
**WHO Mortality Strata**			96.74
A	1.00		99.51
B	1.26 (0.92, 1.72)	0.147	99.38
D	0.99 (0.65, 1.50)	0.147	98.50
**Countries**			96.41
Australia	1.00		99.51
India	1.15 (0.67, 1.96)	0.611	- ^Δ^
Malaysia	1.28 (0.91, 1.81)	0.152	99.34
Philippines	1.34 (0.85, 2.10)	0.196	- ^Δ^
**Year of data collection**			96.74
1981–2000	1.00		98.91
2001–2020	0.98 (0.82, 1.19)	0.891	99.50
**Study Location**			96.73
Community	1.00		99.21
School	1.14 (0.93, 1.40)	0.213	99.68
**Diagnostic method**			96.74
Microscopy	1.00		99.39
Serology	0.63 (0.57, 0.70)	0.000	- ^Δ^
**Number of Infections**			93.88
Single	1.00		98.65
Multiple	1.01 (0.90, 1.14)	0.808	98.95
**QA Grade**			96.73
Low	1.00		97.01
Medium	0.99 (0.73, 1.33)	0.920	99.33
High	1.18 (0.75, 1.86)	0.470	99.87

Note: Bivariate meta-regression analysis was only undertaken where there were 3 or more data sets

^α^ the variation in effect size attributable to heterogeneity

^Δ^ I^2^ not calculated where degrees of freedom ≤3

**Fig 3:**
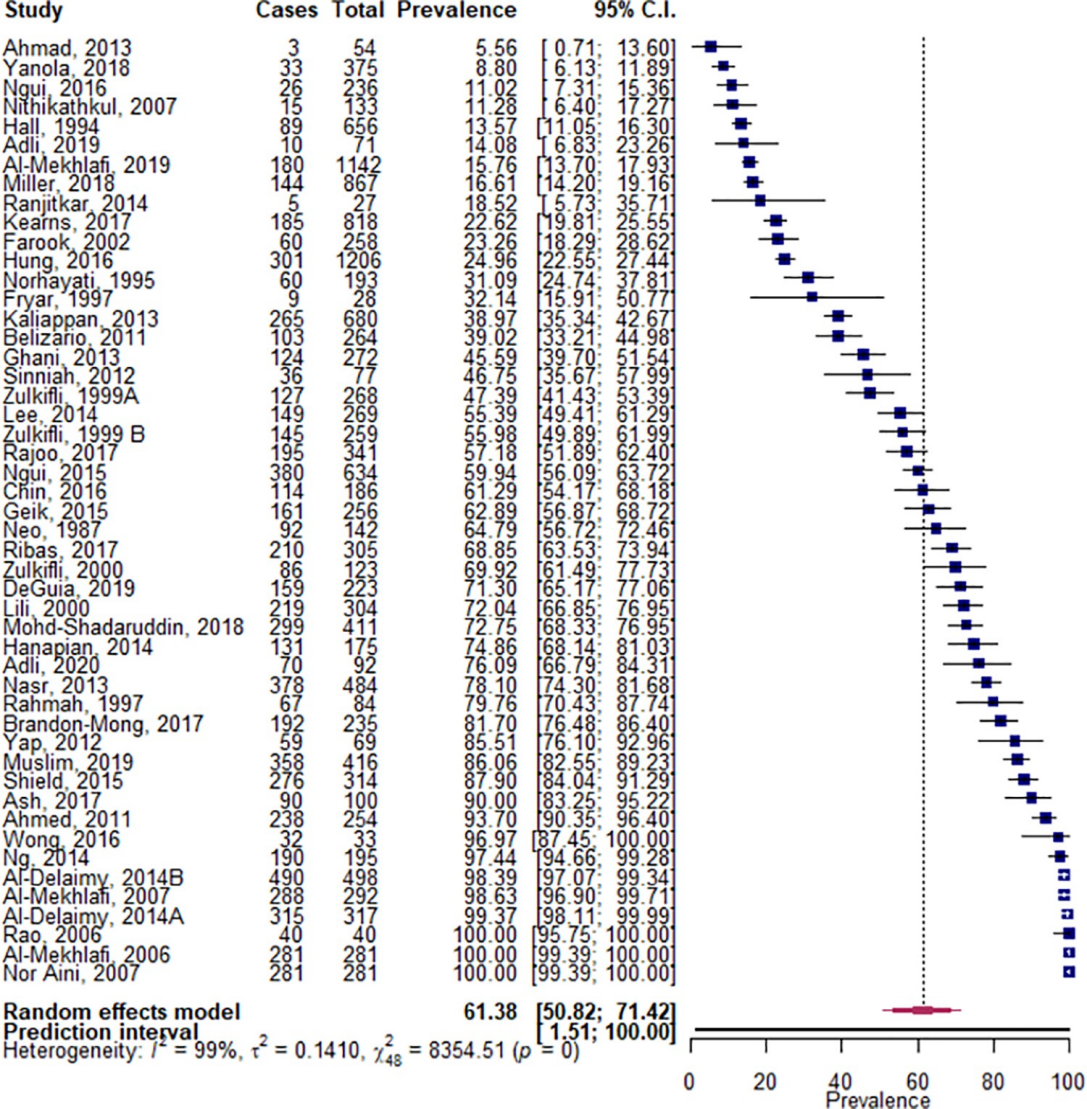
Pooled prevalence of STH infections within minority indigenous study populations. The forest plot shows the pooled prevalence of STH infection with 95% confidence intervals (CI) and the prediction interval. The I^2 statistic is rounded to the nearest integer.

The pooled prevalence of STH infection across the 49 studies, which represented 15,238 minority indigenous participants, was 61.4% (95% CI 50.8, 71.4), with high (I^2^ = 99.4%) and significant (*p* = 0.000) heterogeneity shown between studies (Fig 3). Eighty-six percent of the studies were undertaken in the WPR and 61% of the studies that reported overall STH prevalence, had been undertaken within Malaysia. The prevalence of infection was found to be significantly higher in the WPR at 66.3% (95% CI 55.2, 76.6) compared to the SEAR at 30.3% (95% CI 15.6, 47.3; *p* = 0.010). The only other study covariate found to have a significant effect on overall STH prevalence, was the use of serology as a diagnostic method relative to microscopy (*p* = 0.000). Where studies detailed the number of infections, the prevalence of single and multiple species infections were found to be comparable.

Five studies provided data that could be used to compare STH infection prevalence between minority indigenous and other population groups. Although the prevalence of infection was higher in minority indigenous populations (41.9%, 95% CI 15.6, 70.9) relative to other groups (37.5%, 95% CI 10.6, 69.5) this was not found to be significant (*p* = 0.870).

### Prevalence of *Ascaris lumbricoides* infection

Out of the 81 studies, 64 reported on the prevalence of *A*. *lumbricoides* infection. Details on the pooled prevalence of infection and bivariate meta-regression across the study covariates are detailed in Tables 4 and 5, respectively.

**Table 4:** Pooled prevalence of *A*.*lumbricoides* infections analysed by study covariates.

Categories	Pooled prevalence of *A*.*lumbricoides*^∞^ Infection	
	Studies (n)	Pooled Prevalence (95% CI)
**Population group**		
Minority indigenous populations	64	32.33 (25.72, 39.30)
**Comparative Studies**		
Non-minority indigenous populations	8	25.22 (8.41, 47.20)
Minority indigenous populations	8	41.01 (25.73, 57.21)
	**Analysis on minority indigenous populations only**	
**WHO regions**		
SEAR	19	16.46 (8.22, 26.76)
WPR	45	39.82 (31.98, 47.92)
**WHO Mortality Strata**		
A	1	NA
B	52	34.39 (27.21, 41.95)
D	11	17.66 (6.50, 32.61)
**Countries**		
China	4	67.75 (38.95, 90.70)
India	11	17.66 (6.50, 32.61)
Indonesia	2	26.00 (22.95, 29.18)
Japan	1	NA
Laos	2	10.64 (7.78, 13.87)
Malaysia	32	38.26 (31.79, 44.94)
Philippines	3	44.72 (9.67, 83.17)
Solomon Islands	1	NA
Thailand	6	13.61 (3.79, 27.99)
Vietnam	2	27.13 (25.63, 28.66)
**Year of data collection**		
1949–1980	5	38.96 (2.50, 85.84)
1981–2000	18	29.78 (19.10, 41.69)
2001–2020	41	32.65 (24.45, 41.42)
**Study Location**		
Community	46	33.11 (25.61, 41.06)
School	18	30.37 (17.77, 44.66)
**Sex**		
Male	10	33.66 (22.06, 46.32)
Female	10	34.63 (22.60, 47.72)
**QA Grade**		
Low	8	39.37 (13.21, 69.30)
Medium	53	30.48 (12.51, 37.93)
High	3	47.35 (42.95, 51.77)

Notes

^∞^ Where studies report *Ascaris* infection in humans, data is classified as *A*.*lumbricoides*.

**Table 5:** Bivariate meta-regression of *A*.*lumbricoides* infections analysed by study covariates.

Categories	Pooled prevalence of *A*.*lumbricoides* Infection		
	95% CI	*p*-value	I^2^ ^α^ (%)
**Comparative Studies**			95.77
Non-minority indigenous populations	1.00		99.47
Minority indigenous populations	1.13 (0.86, 1.49)	0.86	98.21
**Analysis on minority indigenous populations only**			
**WHO regions**			93.59
SEAR	1.00		99.10
WPR	1.23 (1.08, 1.40)	0.002	98.92
**WHO Mortality Strata**			94.23
B	1.00		98.98
D	0.86 (0.73, 1.02)	0.077	99.23
**Countries**			94.56
Thailand	1.00		98.06
China	1.63 (1.20, 2.20)	0.002	98.53
India	1.05 (0.84, 1.34)	0.678	99.23
Malaysia	1.26 (1.03, 1.53)	0.022	97.46
Philippines	1.32 (0.88, 1.96)	0.171	- ^Δ^
**Year of data collection**			94.43
1949–1980	1.00		99.59
1981–2000	0.91 (0.63, 1.32)	0.616	99.14
2001–2020	0.92 (0.64, 1.32)	0.651	99.03
**Study Location**			94.33
School	1.00		99.18
Community	1.03 (0.89, 1.18)	0.712	99.06
**Sex**			77.10
Male	1.00		94.60
Female	1.01 (0.84, 1.22)	0.898	95.25
**QA Grade**			94.55
Low	1.00		99.28
Medium	0.93 (0.73, 1.18)	0.532	33.13
High	1.06 (0.84, 1.35)	0.601	- ^Δ^

Note: Bivariate meta-regression analysis was only undertaken where there were 3 or more data sets

^α^ the variation in effect size attributable to heterogeneity

^Δ^ I^2^ not calculated where degrees of freedom ≤3

**Fig 4:**
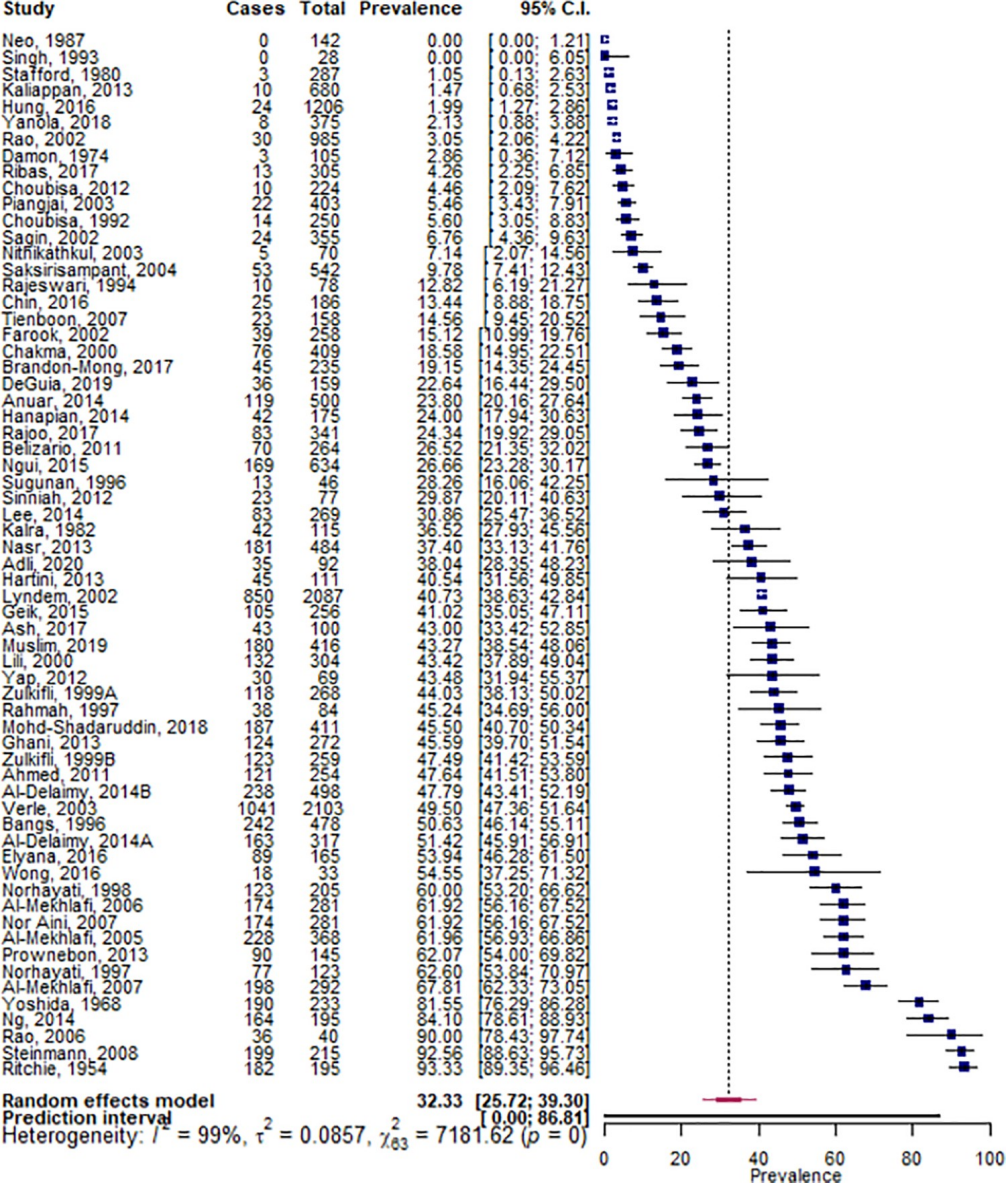
Pooled prevalence of *A*.*lumbricoides* infections within minority indigenous study populations. The forest plot shows the pooled prevalence of *A*.*lumbricoides* infection with 95% confidence intervals (CI) and the prediction interval. The I^2 statistic is rounded to the nearest integer.

The pooled prevalence of *A*. *lumbricoides* infection across the 64 studies, representing 21,495 minority indigenous participants, was 32.3% (95% CI 25.7, 39.3- Fig 4). Although there was significant heterogeneity between publications, the only study covariates of significance were WHO region and country. The WPR, where 70% of the studies were undertaken, had a significantly higher prevalence of infection at 39.8% (95% CI 31.9, 47.9) than the SEAR at 16.5% (95% CI 8.22, 26.8; *p* = 0.002). Where sufficient data were available to allow the country of study to be analyzed as a covariate, prevalence was found to be significantly higher in China (67.8%, 95% CI 39.0, 90.7; *p* = 0.002) and Malaysia (38.3%, 95% CI 31.8, 44.9; *p* = 0.022) than elsewhere.

Eight studies provided data that facilitated a comparison of *A*.*lumbricoides* infection prevalence between minority indigenous and other population groups. Although not significant (*p* = 0.860), the prevalence of infection was found to be higher in minority indigenous participants (41.0%, 95% CI 25.7, 57.2) compared to those from other population groups (25.2%, 95% CI 8.4, 47.2).

Heterogeneity was found to be high (I^2^ >75%) for *A*.*lumbricoides* prevalence within all covariates, with the exception of QA grade. Studies classified with a medium QA score (5–7) showed moderate heterogeneity (I^2^ 25–75%).

### Prevalence of *Trichuris trichiura* infection

Sixty -five of the 81 studies reported on the prevalence of *T*. *trichiura* infection, representing a cumulative study population of 20,466 minority indigenous participants. The pooled prevalence of infection across the study covariates and the subsequent bivariate meta-regression are detailed in Tables 6 and 7, respectively.

**Table 6:** Pooled prevalence of *T*.*trichiura* infections analysed by study covariates.

Categories	Pooled prevalence of *T*.*trichiura*^∞^ Infection	
	Studies (n)	Pooled Prevalence (95% CI)
**Population group**		
Minority indigenous populations	65	43.55 (32.62, 54.80)
**Comparative Studies**		
Non-minority indigenous populations	8	24.64 (15.49, 35.11)
Minority indigenous populations	8	42.52 (26.93, 58.91)
	**Analysis on minority indigenous populations only**		
**WHO regions**		
SEAR	16	10.33 (5.21, 16.85)
WPR	49	55.82 (44.21, 67.12)
**WHO Mortality Strata**		
A	6	29.36 (1.58, 71.54)
B	51	49.65 (38.13, 61.20)
D	8	16.51 (6.31, 30.10)
**Countries**		
Australia	5	26.65 (0.00, 78.48)
China	4	51.61 (12.98, 89.12)
India	8	16.51 (6.31, 30.10)
Indonesia	2	10.52 (8.43, 12.80)
Japan	1	NA
Laos	2	30.55 (26.13, 35.15)
Malaysia	31	67.82 (56.71, 77.99)
Philippines	3	40.03 (0.11, 93.81)
Solomon Islands	1	NA
Thailand	6	5.70 (4.23, 7.37)
Vietnam	2	23.92 (22.48, 25.39)
**Year of data collection**		
1949–1980	5	17.61 (3.98, 37.85)
1981–2000	20	33.78 (18.08, 51.54)
2001–2020	40	52.07 (36.98, 66.96)
**Study Location**		
Community	48	40.28 (28.72, 52.40)
School	17	52.92 (26.20, 78.79)
**Sex**		
Male	10	55.15 (31.91, 77.30)
Female	10	53.97 (30.52, 76.54)
**QA Grade**		
Low	10	25.14 (11.56, 41.74)
Medium	52	43.97 (31.94, 56.36)
High	3	91.61 (71.62, 99.99)

Notes

^∞^ Where studies report *Trichuris* infection in humans, data is classified as *T*. *trichiura*.

**Table 7:** Bivariate meta-regression of *T*. *trichiura* infections analysed by study covariates.

Categories	Pooled prevalence of *T*.*trichiura* Infection		
	95% CI	*p* value	I^2^ ^α^ (%)
**Comparative Studies**			90.27
Non-minority indigenous populations	1.00		97.85
Minority indigenous populations	1.19 (0.95, 1.48)	0.115	98.25
**Analysis on minority indigenous populations only**			
**WHO regions**			95.80
SEAR	1.00		97.85
WPR	1.48 (1.28, 1.72)	0.000	99.49
**WHO Mortality Strata**			96.97
A	1.00		99.49
B	1.17 (0.89, 1.52)	0.253	99.54
D	0.90 (0.65, 1.26)	0.540	98.32
**Countries**			97.33
Thailand	1.00		39.85
Australia	1.31 (0.95, 1.79)	0.097	99.58
China	1.57 (1.08, 2.29)	0.018	99.33
India	1.20 (0.95, 1.52)	0.120	98.32
Malaysia	1.80 (1.62, 2.00)	0.000	99.09
Philippines	1.41 (0.86, 2.30)	0.169	- ^Δ^
**Year of data collection**			97.13
1949–1980	1.00		97.95
1981–2000	1.18 (0.97, 1.43)	0.092	99.55
2001–2020	1.38 (1.17, 1.63)	0.000	99.64
**Study Location**			97.38
Community	1.00		99.56
School	1.11 (0.90, 1.37)	0.303	99.74
**Sex**			91.30
Male	1.00		98.48
Female	1.00 (0.73, 1.37)	0.991	98.56
**QA Grade**			97.03
Low	1.00		98.28
Medium	1.18 (0.97, 1.43)	0.094	99.62
High	1.81 (1.46, 2.25)	0.000	- ^Δ^

Note: Bivariate meta-regression analysis was only undertaken where there were 3 or more data sets

^α^ the variation in effect size attributable to heterogeneity

^Δ^ I^2^ not calculated where degrees of freedom ≤3

**Fig 5:**
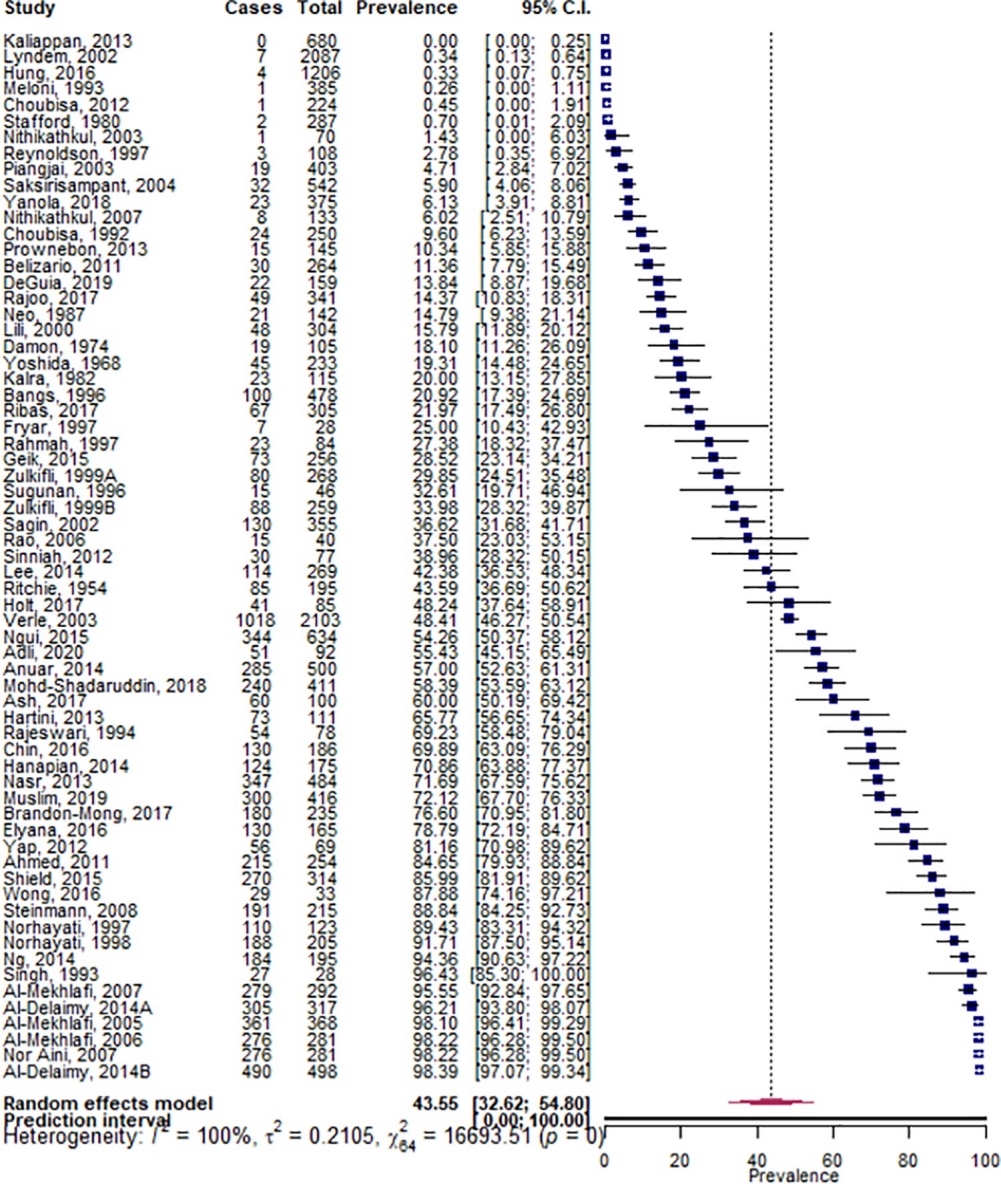
Pooled prevalence of *T*.*trichiura* infections within minority indigenous study populations. The forest plot shows the pooled prevalence of *T*.*trichiura* infection with 95% confidence intervals (CI) and the prediction interval. The I^2 statistic is rounded to the nearest integer.

The pooled prevalence of *T*. *trichiura* infection within minority indigenous populations was 43.6% (95% CI 32.6, 54.8- Fig 5). There was significant heterogeneity between studies, with WHO region, country of study, period of data collection, and QA grade shown to be significant study co-variates. The prevalence of infection was shown to be significantly higher in the WPR at 55.8% (95% CI 44.2, 67.1) compared to the SEAR at 10.3% (95% CI 5.2, 16.9; *p* = 0.000). Where sufficient data were available to evaluate the country of study as a covariate, infection prevalence was significantly higher in China (51.6%, 95% CI 13.0, 89.1; *p* = 0.018) and Malaysia (67.8%, 95% CI 56.7, 78.0; *p* = 0.000). *T*. *trichiura* infection was found to be significantly higher in 2001–2020 (52.1%, 95% CI 37.0, 67.0) compared to 1949–1980 (17.6%, 95% CI 4.0, 37.9; *p* = 0.000). High QA grade studies were shown to have a significantly higher prevalence of *T*. *trichiura* infection (91.6%, 95% CI 71.62, 99.99) than low QA grade studies (25.1%, 95% CI 11.56, 41.74).

Eight studies reported data that facilitated a comparison of infection prevalence between minority indigenous and other population groups. Although the differential in *T*. *trichiura* prevalence was not significant (*p* = 0.115), it was higher in minority indigenous study participants (42.5%, 95% CI 26.9, 58.9) in comparison to those from other population groups (24.6%, 95% CI 15.5, 35.1).

### Prevalence of hookworm infection

Sixty-eight studies presented data on hookworm infection, representing a cumulative minority indigenous study population of 21,967 participants. The pooled prevalence of infection across the study covariates and the subsequent bivariate meta-regression are detailed in Tables 8 and 9, respectively.

**Table 8:** Pooled prevalence of Hookworm infections analysed by study covariates.

Categories	Pooled prevalence of Hookworm^∞^ Infection	
	Studies (n)	Pooled Prevalence (95% CI)
**Population group**		
Minority indigenous populations	68	19.92 (15.68, 24.53)
**Comparative Studies**		
Non-minority indigenous populations	8	10.69 (1.56, 26.27)
Minority indigenous populations	8	16.73 (3.93, 35.67)
	**Analysis on minority indigenous populations only**		
**WHO regions**		
SEAR	17	17.75 (10.20, 26.80)
WPR	51	20.66 (15.55, 26.28)
**WHO Mortality Strata**		
A	6	7.80 (0.00, 25.42)
B	52	21.42 (16.21, 27.14)
D	10	20.35 (12.68, 29.26)
**Countries**		
Australia	5	10.87 (0.12, 32.75)
China	4	49.84 (20.84, 78.90)
India	10	20.35 (12.68, 29.26)
Indonesia	2	50.05 (46.50, 53.59)
Japan	1	NA
Laos	2	61.53 (56.71, 66.23)
Malaysia	33	17.18 (13.25, 21.51)
Philippines	3	15.95 (11.18, 21.37)
Solomon Islands	1	NA
Thailand	5	5.53 (1.91, 10.72)
Vietnam	2	40.71 (39.04, 42.39)
**Year of data collection**		
1949–1980	5	29.45 (7.27, 58.68)
1981–2000	22	20.86 (13.65, 29.11)
2001–2020	41	18.38 (13.44, 23.89)
**Study Location**		
Community	52	21.17 (15.95, 26.90)
School	16	15.90 (10.77, 21.79)
**Sex**		
Male	13	19.06 (13.67, 25.08)
Female	13	16.58 (11.57, 22.27)
**Hookworm Species**		
*A*.*duodenale*	5	11.56 (1.27, 29.68)
*N*.*americanus*	4	44.93 (23.83, 67.04)
*A*.*ceylanicum*	1	NA
**QA Grade**		
Low	11	17.29 (5.61, 33.36)
Medium	54	20.06 (15.37, 25.18)
High	3	27.56 (20.98, 34.66)

Notes

^∞^ Where studies reported by species, figures were aggregated to give overall hookworm prevalence which was evaluated against the study co-variates with the exception of the covariate ‘hookworm species’

**Table 9:** Bivariate meta-regression of Hookworm infections analysed by study covariates.

Categories	Pooled prevalence of Hookworm Infection		
	95% CI	*p* value	I^2^ ^α^ (%)
**Comparative Studies**			94.43
Non-minority indigenous populations	1.00		99.33
Minority indigenous populations	1.06 (0.85, 1.32)	0.597	99.01
**Analysis on minority indigenous populations only**			
**WHO regions**			90.85
SEAR	1.00		98.72
WPR	1.02 (0.92, 1.14)	0.659	98.45
**WHO Mortality Strata**			90.78
A	1.00		98.33
B	1.12 (0.98, 1.29)	0.100	98.57
D	1.12 (0.95, 1.32)	0.188	97.77
**Countries**			88.13
Thailand	1.00		90.08
Australia	1.08 (0.92, 1.28)	0.329	98.28
China	1.55 (1.12, 2.15)	0.009	98.68
India	1.18 (1.04, 1.33)	0.010	97.77
Malaysia	1.13 (1.05, 1.21)	0.001	96.04
Philippines	1.10 (1.02, 1.18)	0.014	- ^Δ^
**Year of data collection**			90.01
1949–1980	1.00		98.80
1981–2000	0.90 (0.73, 1.10)	0.301	98.70
2001–2020	0.88 (0.72, 1.07)	0.186	98.13
**Study Location**			90.14
Community	1.00		98.66
School	0.94 (0.86, 1.02)	0.127	96.27
**Sex**			28.46
Male	1.00		89.38
Female	0.98 (0.90, 1.06)	0.580	89.75
**QA Grade**			90.78
Low	1.00		98.51
Medium	1.01 (0.88, 1.16)	0.856	98.56
High	1.06 (0.93, 1.22)	0.377	- ^Δ^

Note: Bivariate meta-regression analysis was only undertaken where there were 3 or more data sets

^α^ the variation in effect size attributable to heterogeneity

^Δ^ I^2^ not calculated where degrees of freedom ≤3

**Fig 6:**
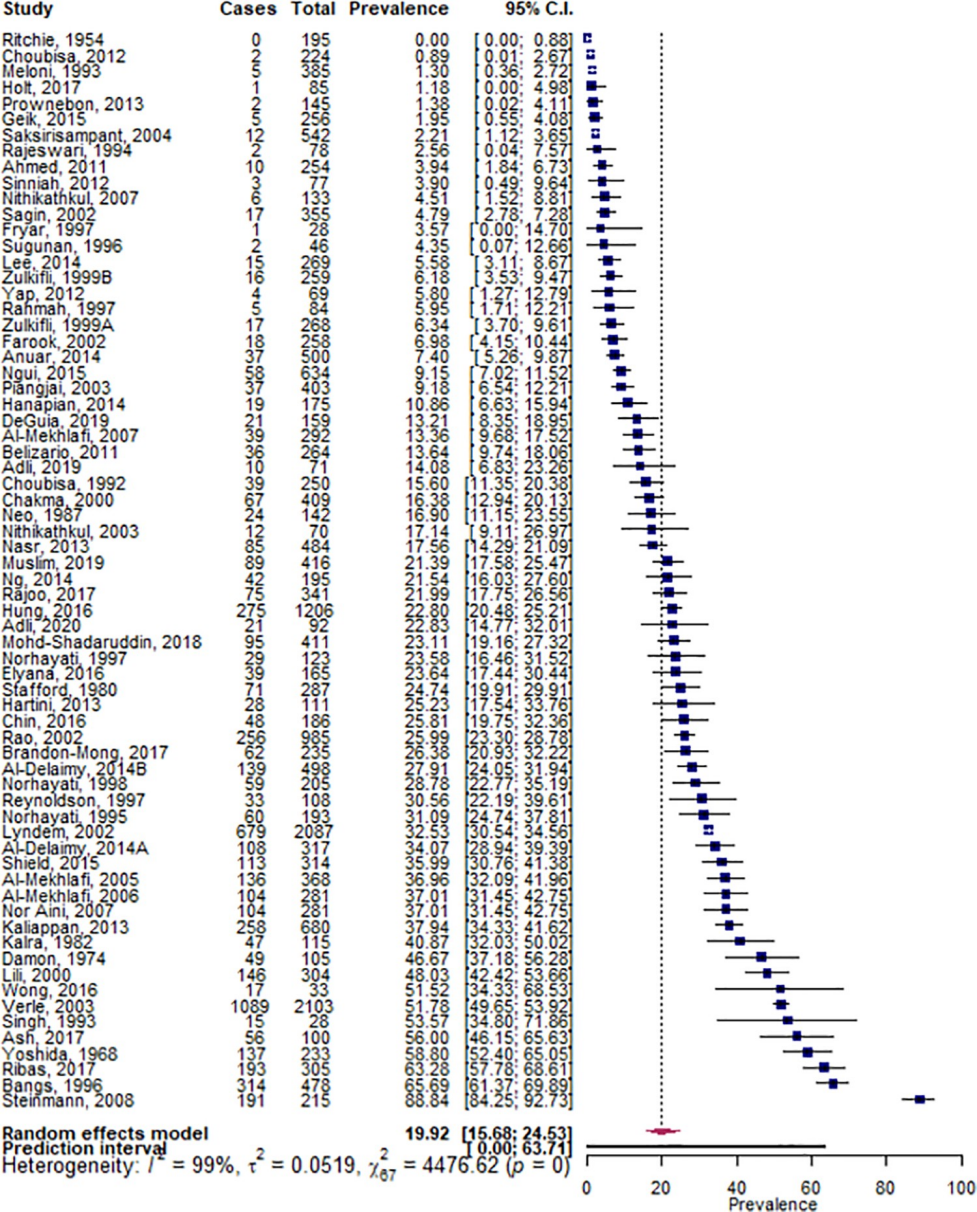
Pooled prevalence of hookworm infections within minority indigenous study populations. The forest plot shows the pooled prevalence of hookworm infection with 95% confidence intervals (CI) and the prediction interval. The I^2 statistic is rounded to the nearest integer.

The pooled prevalence of hookworm infection was 19.9% (95% CI 15.7, 24.5) within minority indigenous populations (Fig 6). The heterogeneity between studies was found to be high (I^2^ = 98.5%) and significant (*p* = 0.000). The country of study was found to be the only significant study covariate and although there were insufficient data to evaluate all countries represented, four countries were found to have a significantly higher prevalence of infection than other countries. These countries were: China (49.8%, 95% CI 20.8, 78.9; *p* = 0.009), India (20.4%, 95% CI 12.7, 29.3; *p* = 0.010), Malaysia (17.2%, 95% CI 13.3, 21.5; *p* = 0.001)) and the Philippines (16.0%, 95% CI 11.2, 21.4; *p* = 0.014).

Eight studies detailed the species of hookworm they identified. Based on these publications, *N*.*americanus* was more prevalent (44.9%, 95% CI 23.8, 67.0) than *A*. *duodenale* (11.6%, 95% CI 1.3, 29.7) and *A*.*ceylanicum* was reported in one study only.[32] In addition to these eight studies, three studies[33–35] undertook further analysis on a subset of their hookworm positive samples and identified *N*. *americanus*, *A*. *duodenale*, *A*. *ceylanicum* and *Anclostoma brazilienze*.

Eight studies presented data that enabled a comparison of hookworm infection prevalence to be evaluated between minority indigenous and other populations. Although the difference between population groups was not found to be significant (*p* = 0.597), it was higher in minority indigenous participants (16.7%, 95% CI 3.9, 35.7) than those from other population groups (10.7%, 95% CI 1.6, 26.3).

### Prevalence of *Strongyloides stercoralis* infection

Twenty studies over a cumulative 7,020 minority indigenous participants reported on the prevalence of *S*.*stercorlais* infection. The prevalence of infection analyzed by study co-variates is detailed in Table 10 and the subsequent meta-analysis in Table 11.

**Table 10:** Pooled prevalence of *S*. *stercoralis* infections analysed by study covariates.

Categories	Pooled prevalence of *S*. *stercoralis* ^∞^ Infection	
	Studies (n)	Pooled Prevalence (95% CI)
**Population group**		
Minority indigenous populations	20	6.26 (3.16, 10.24)
**Comparative Studies**		
Non-minority indigenous populations	1	NA
Minority indigenous populations	1	NA
	**Analysis on minority indigenous populations only**		
**WHO regions**		
SEAR	6	4.00 (0.35, 10.55)
WPR	14	7.35 (3.64, 12.14)
**WHO Mortality Strata**		
A	7	8.10 (2.17, 17.03)
B	10	4.98 (1.61, 9.92)
D	3	6.79 (0.01, 21.40)
**Countries**		
Australia	7	8.10 (2.17, 17.03)
Bangladesh	1	NA
China	1	NA
India	2	0.93 (0.00, 2.88)
Indonesia	1	NA
Laos	1	NA
Malaysia	5	6.11 (1.08, 14.42)
Thailand	2	1.11 (0.33, 2.21)
**Year of data collection**		
1981–2000	8	4.63 (0.55, 11.65)
2001–2020	12	7.39 (3.41, 12.66)
**Study Location**		
Community	18	6.25 (2.95, 10.58)
School	2	9.22 (7.88, 10.66)
**Diagnostic method** *		
Microscopy	15	4.14 (1.58, 7.68)
PCR	2	14.95 (12.95, 17.06)
Serology	3	16.78 (11.43, 22.92)
**Sex**		
Male	3	18.61 (15.77, 21.61)
Female	3	4.07 (0.00, 14.29)
**QA Grade**		
Low	3	2.45 (0.00, 10.73)
Medium	15	6.94 (3.25, 11.79)
High	2	10.77 (9.27, 12.36)

Notes

^∞^ Human *strongyloides* infection classified as *S*.*stercoralis*

*Diagnostic method: PCR and microscopy classified as PCR; ELISA classified as serology

**Table 11:** Bivariate meta-regression of *S*. *stercoralis* infections analysed by study covariates.

Categories	Pooled prevalence of *S*.*stercoralis* ^∞^ Infection		
	CI 95%	*p* value	I^2^ ^α^ (%)
**Analysis on minority indigenous populations only**			
**WHO regions**			49.02
SEAR	1.00		96.37
WPR	1.03 (0.97, 1.10)	0.287	96.62
**WHO Mortality Strata**			53.54
A	1.00		97.56
B	0.96 (0.88, 1.04)	0.316	96.43
D	0.98 (0.88, 1.09)	0.706	- ^Δ^
**Countries**			47.25
Malaysia	1.00		96.24
Australia	1.03 (0.91, 1.16)	0.590	97.56
**Year of data collection**			55.70
1981–2000	1.00		96.58
2001–2020	1.02 (0.95, 1.10)	0.507	97.18
Diagnostic method *			55.28
Microscopy	1.00		94.90
Serology	1.12 (1.04, 1.20)	0.004	-^Δ^
**Sex**			0.000
Male	1.00		- ^Δ^
Female	0.88 (0.78, 0.99)	0.046	- ^Δ^
**QA Grade**			56.30
Low	1.00		- ^Δ^
Medium	1.05 (0.99, 1.11)	0.091	96.89

Note: Bivariate meta-regression analysis was only undertaken where there were 3 or more data sets

^∞^ Human *strongyloides* infection classified as *S*.*stercoralis*

^α^ the variation in effect size attributable to heterogeneity

^Δ^ I^2^ not calculated where degrees of freedom ≤3

**Fig 7:**
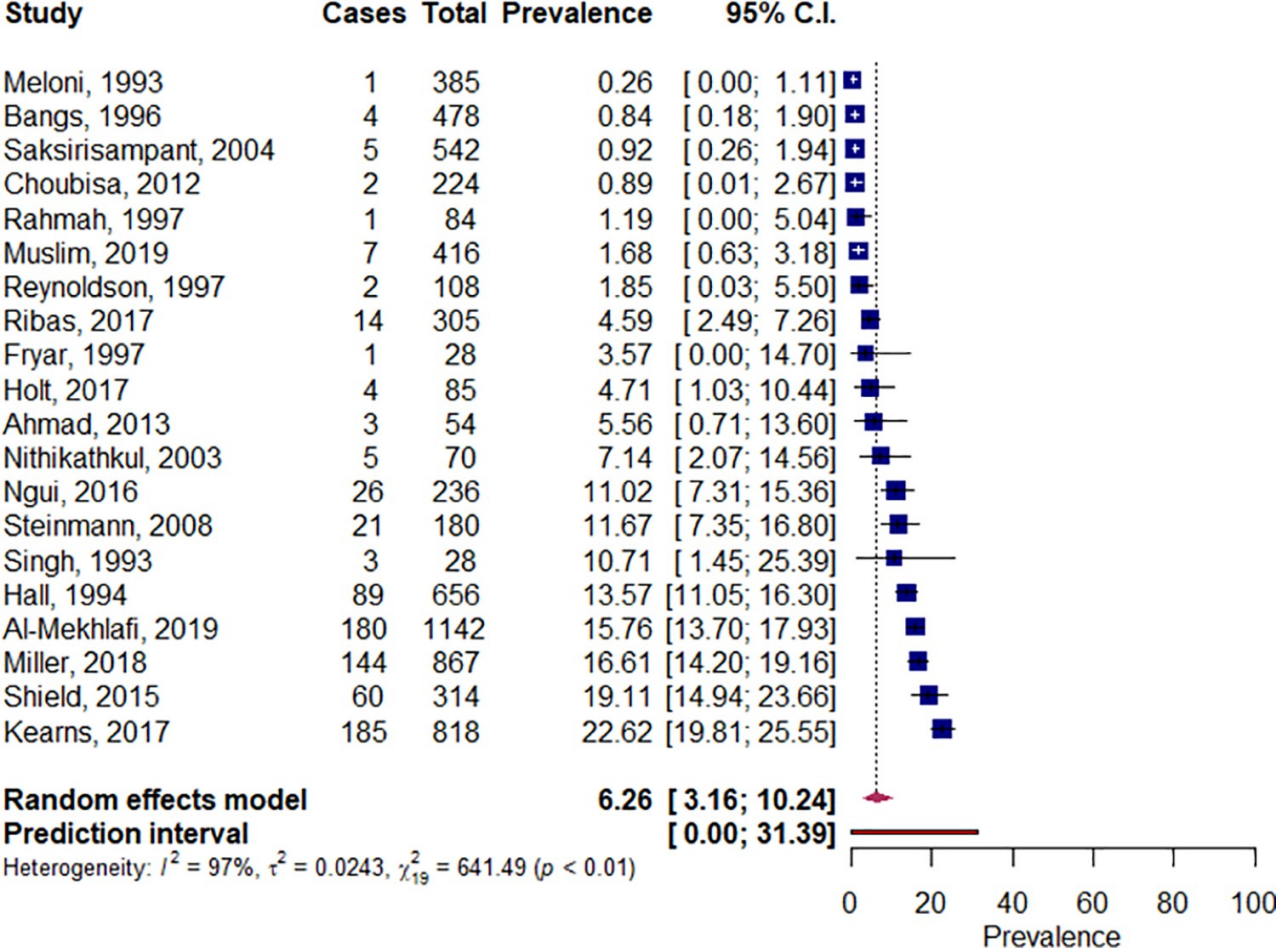
Pooled prevalence of *S*.*stercoralis* infections within minority indigenous study populations. The forest plot shows the pooled prevalence of *S*.*stercoralis* infection with 95% confidence intervals (CI) and the prediction interval. The I^2 statistic is rounded to the nearest integer.

The pooled prevalence of infection within minority indigenous populations was 6.3% (95% CI 3.2, 10.2) with a high and significant degree of heterogeneity between studies (Fig 7). From the study co-variates analyzed, diagnostic method and sex where the only two covariates to demonstrate a significant association with infection prevalence. Disease prevalence was significantly higher when serology was used as a diagnostic (16.8%, 95% CI 11.4, 22.9) compared to microscopy (4.1%, 95% CI 1.6, 7.7; *p* = 0.004). Females had a significantly lower prevalence of infection (4.1%, 95% CI 0.0, 14.3) compared to males (18.6%, 15.8, 21.6; *p* = 0.046).

There was only one study that provided data enabling a comparison of *S*. *stercorlais* prevalence between minority indigenous and other population participants. Although it was not possible to evaluate the significance of the results, it is noted that prevalence was higher in minority indigenous participants (13.6%, 95% CI 11.2, 16.4) compared to those in other population groups (5.1%, 95% CI 2.8, 9.1).

## Discussion

The systematic review shows a high prevalence of STH infection amongst minority indigenous populations. It is likely that the true prevalence of infection is higher due to the low sensitivity of diagnostic methods used.[36] This potential under-estimation of infection is particularly likely in minority indigenous communities, for whom the provision of faecal samples presents a significant obstacle due to cultural beliefs, thereby creating a challenge to the recommended serial sampling over multiple days.[37,38]

The results from our review show the prevalence of infection to be consistently higher in the WPR than the SEAR, although WHO figures show the DALYs to be higher overall within the SEAR.[6] Although there are many potential confounders, the higher prevalence of infection within the WPR identified by this review may reflect a higher burden of disease within indigenous minority populations in this region.

Although research shows the prevalence and intensity of STH infection to be related to socioeconomic status and hygiene conditions,[39–43] it is interesting to note that the review found no significant difference in disease prevalence for some STH between countries that have very different socio-economic profiles. For example, the review shows there to be no significant difference in overall STH infection in minority indigenous populations between Australia, which in 2020 ranked eighth on the Human Development Index (HDI), and India which ranked 131^st^. [44] This re-enforces the fact that vulnerable population groups within otherwise highly developed countries continue to be at risk of NTDs such as STH infection.

Although it is hoped that economic development and preventative chemotherapy programs have led to a reduction in the global burden of STH infection over time,[18] results from the systematic review show the prevalence of overall STH infections within minority indigenous populations to have remained static. When the review analyzes the prevalence of infection by species, some interesting trends are observed. In particular, the prevalence. of *S*. *stercorlais* and *T*. *trichiura* infections within minority indigenous populations have increased over time, with the increasing prevalence of *T*. *trichiura* being significant. The increasing trend in *S*. *stercoralis* prevalence may in part be due to developments in diagnostic capabilities as the parasite is very difficult to detect by microscopy;[45] but may also reflect the treatment challenges presented by its autoinfection capability.[46] The significant increase in *T*.*trichiura* infection within this vulnerable population group however warrants further investigation. Although the WHO recommend the administration of albendazole or mebendazole as part of their STH control strategy,[2] these drugs are shown to have limited efficacy against *T*. *trichiura*. [47,48]

Although the review provides an indication of STH prevalence within indigenous minority populations as a collective, research showing the significant heterogeneity in infection prevalence and intensity between individuals within a population is noted. [36] There is an argument that infection intensity would be a more useful metric than prevalence, as morbidity severity is relative to infection intensity and heavily infected individuals present a major source of infection for their community.[36]

If the 2021–2030 NTD road map targets[49] are to be achieved, countries need to address the impact of STH infections within their vulnerable indigenous populations. By impacting productivity and human development, STH infections re-enforce poverty,[50] which already disproportionately affects these communities[19]. To be effective, interventions need to be culturally appropriate[51] and as a result of disruptions to public health programmes caused by the Coronavirus Disease 2019 (COVID 19) pandemic, they will need to be increasingly innovative if 2021–2030 targets are to be achieved.[52]

This systematic review provided information on STH prevalence amongst minority indigenous populations of the SEAR and WPRs and showed where further data and research are required. However, the limitations of systematic reviews and the scope of data need to be taken into consideration when results of the systematic review are used to inform public health policy. The following limitations of the review are noted. Publication bias is an inherent potential limitation of the systematic review process. As a result of resource constraints data extraction was limited to articles published in English. The accuracy of estimating disease prevalence may be impacted by the inclusion of small study populations. The review did not take into consideration the effect of treatment and intervention regimes which may impact infection prevalence over time. The definition of a minority indigenous population is not based upon a universal classification.

## Conclusion

STH infections continue to create a significant global health burden within vulnerable communities. Soil transmitted helminthiasis is prevalent within indigenous communities who reside in countries across the spectrum of WHO mortality strata. To stop the ongoing impacts of STH infection upon the poverty cycle, accurate relevant prevalence and infection intensity data are required to inform innovative and culturally appropriate interventions.

## Supporting information

S1 PRISMA Checklist (DOCX)

S1 Table Systematic review search terms summary. (DOCX)

S2 Table QA assessment of STH studies based on modified Newcastle-Ottawa Quality Assessment Scale. (DOCX)

S3 Table Key to modified Newcastle-Ottawa Quality Assessment Scale scoring. (DOCX)
