# Supplementary material for: The prevalence of soil transmitted helminth infections in minority indigenous populations of South-East Asia and the Western Pacific Region: A systematic review and meta-analysis
Source: PLoS Negl Trop Dis. 2021 Nov 10;15(11):e0009890. doi: 10.1371/journal.pntd.0009890 (PMC8580241; doi:10.1371/journal.pntd.0009890)
Supplement: S1 Table — (DOCX) [file pntd.0009890.s002.docx]

S1 Table B: Systematic review search terms summary

| **Descriptor** | **Search Terms** |
| --- | --- |
| STH search terms | *“soil transmitted helminth*”* OR *STH* OR *Ascaris* OR *Trichuris* OR *Nectator* OR *Ancylostoma* OR *Strongyloides* OR *hookworm** **AND** |
| ^∆^ Countries within the SEAR and WPR | *Indonesia* OR “*Sri Lanka*” OR *Ceylon* OR *Thailand* OR *Timor** OR *Bangladesh* OR *Bhutan* OR “*Democratic People’s Republic of Korea*” OR *India* OR *Maldives* OR *Myanmar* OR *Burma* OR *Nepal* OR *Australia* OR *Brunei* OR *Japan* OR “New Zealand” OR Cambodia OR China OR “*Cook Islands*” OR *Fiji* OR *Kiribati* OR *Lao** OR *Malaysia* OR “*Marshall Islands*” OR *Micronesia* OR Mongolia OR Nauru OR Niue OR Palau OR “*Papua New Guinea*” OR *Philippines* OR “*Republic of Korea*” OR *Samoa* OR “*Solomon Islands*” OR *Tonga* OR *Tuvalu* OR *Vanuatu* OR *Vietnam* **AND** |
| ^α^ Indigenous search terms | *Indigenous* OR *aborigin** OR *native* OR *“first nation*”* OR *“ethnic group”* OR *tribal* OR *tribe* OR *autochthonous* |

^∆^ The WHO Global Burden of Disease (GBD) regional classification system^[1]^ was used to define the countries located within the SEAR and WPR. Singapore was omitted from the search as there are no minority indigenous populations according to the classification criteria used in this review.

^α^ In addition to the above generic indigenous search terms, those relevant to each country were included. The country specific search terms were derived from the International Working Group on Indigenous Affairs[2], Native Planet- Indigenous Mapping[3], and the World Directory Listing of Minorities and Indigenous People[4]. If indigenous minority study populations were not identified according to the search criteria list, but the author identified them as such, they were included within the analysis.

**References**

1. World Health Organization. Global Burden of Disease Regions used for WHO-CHOICE Analyses n.d. [Available from: <https://www.who.int/choice/demography/regions/en/>.

2. International Work Group for Indigenous Affairs. Who We Are Indigenous Peoples in Asia 2009 [updated 10.03.09. Briefing Paper]. Available from: <https://www.iwgia.org/images/publications/0640_ho_are_e_IPs_in_Asia.pdf>.

3. Native Planet. Indigenous Mapping: Ethnic Communities from Asia n.d. [Available from: <https://www.nativeplanet.org/indigenous/ethnicdiversity/indigenous_data_asia.shtml>.

4. Minority Rights Group International. World Directory of Minorities and Indigenous Peoples n.d. [Available from: <https://minorityrights.org/directory/>.
