# Supplementary material for: The prevalence of soil transmitted helminth infections in minority indigenous populations of South-East Asia and the Western Pacific Region: A systematic review and meta-analysis
Source: PLoS Negl Trop Dis. 2021 Nov 10;15(11):e0009890. doi: 10.1371/journal.pntd.0009890 (PMC8580241; doi:10.1371/journal.pntd.0009890)
Supplement: S2 Table — (DOCX) [file pntd.0009890.s003.docx]

S1 Table C: QA assessment of STH studies based on modified Newcastle-Ottawa Quality Assessment Scale

| Study # | First Author, Year of publication | Study Population | Representativeness of the sample | Ascertainment of specimen collection methods | Sample size | Non-respondents | Impact of Bias (selection bias, measurement bias, participant reporting, confounders) | Assessment of the outcome (STH infection) | Statistical analysis | Total Score | QA Grade |
| --- | --- | --- | --- | --- | --- | --- | --- | --- | --- | --- | --- |
| 1 | Adli, 2019 | 1 | 1 | 1 | 0 | 0 | 0 | 1 | 0 | 4 | low |
| 2 | Adli, 2020 | 1 | 1 | 1 | 0 | 0 | 0 | 1 | 0 | 4 | low |
| 3 | Ahmad, 2013 | 1 | 1 | 1 | 1 | 0 | 1 | 1 | 1 | 7 | medium |
| 4 | Ahmed, 2011 | 1 | 1 | 1 | 0 | 1 | 0 | 1 | 1 | 6 | medium |
| 5 | Al-Delaimy, 2014A | 1 | 1 | 1 | 1 | 1 | 1 | 1 | 1 | 8 | high |
| 6 | Al-Delaimy, 2014B | 1 | 1 | 1 | 1 | 1 | 1 | 1 | 1 | 8 | high |
| 7 | Al-Mekhlafi, 2005 | 1 | 1 | 1 | 0 | 0 | 1 | 1 | 1 | 6 | medium |
| 8 | Al-Mekhlafi, 2006 | 1 | 1 | 1 | 0 | 0 | 0 | 1 | 1 | 5 | medium |
| 9 | Al-Mekhlafi, 2007 | 1 | 1 | 1 | 0 | 1 | 0 | 1 | 1 | 6 | medium |
| 10 | Al-Mekhlafi, 2019 | 1 | 1 | 1 | 1 | 1 | 1 | 1 | 1 | 8 | high |
| 11 | Anuar, 2014 | 1 | 1 | 1 | 0 | 0 | 1 | 1 | 1 | 6 | medium |
| 12 | Ash, 2017 | 1 | 1 | 1 | 0 | 1 | 1 | 1 | 1 | 7 | medium |
| 13 | Bangs, 1996 | 1 | 1 | 1 | 0 | 1 | 1 | 1 | 0 | 6 | medium |
| 14 | Belizario, 2011 | 1 | 1 | 1 | 1 | 0 | 1 | 1 | 1 | 7 | medium |
| 15 | Brandon-Mong, 2017 | 1 | 1 | 1 | 0 | 0 | 0 | 1 | 1 | 5 | medium |
| 16 | Chakma, 2000 | 1 | 1 | 1 | 0 | 1 | 0 | 1 | 0 | 5 | medium |
| 17 | Chin, 2016 | 1 | 1 | 1 | 1 | 0 | 1 | 1 | 1 | 7 | medium |
| 18 | Choubisa, 1992 | 1 | 1 | 1 | 0 | 0 | 0 | 1 | 0 | 4 | low |
| 19 | Choubisa, 2012 | 1 | 1 | 1 | 0 | 0 | 0 | 1 | 1 | 5 | medium |
| 20 | Damon, 1974 | 1 | 1 | 1 | 0 | 1 | 0 | 1 | 0 | 5 | medium |
| 21 | DeGuia, 2019 | 1 | 1 | 1 | 1 | 1 | 0 | 1 | 0 | 6 | medium |
| 22 | Elyana, 2016 | 1 | 1 | 1 | 1 | 0 | 1 | 1 | 1 | 7 | medium |
| 23 | Farook, 2002 | 1 | 1 | 1 | 0 | 1 | 0 | 1 | 1 | 6 | medium |
| 24 | Fryar, 1997 | 1 | 1 | 1 | 0 | 0 | 0 | 1 | 0 | 4 | low |
| 25 | Geik, 2015 | 1 | 1 | 1 | 1 | 0 | 0 | 1 | 1 | 6 | medium |
| 26 | Ghani, 2013 | 1 | 1 | 1 | 0 | 1 | 0 | 1 | 0 | 5 | medium |
| 27 | Hall, 1994 | 1 | 1 | 1 | 0 | 0 | 1 | 1 | 1 | 6 | medium |
| 28 | Hanapian, 2014 | 1 | 1 | 1 | 0 | 1 | 0 | 1 | 1 | 6 | medium |
| 29 | Hartini, 2013 | 1 | 1 | 1 | 0 | 1 | 0 | 1 | 1 | 6 | medium |
| 30 | Holt, 2017 | 1 | 1 | 1 | 0 | 0 | 1 | 1 | 1 | 6 | medium |
| 31 | Hung, 2016 | 1 | 1 | 1 | 0 | 0 | 0 | 1 | 1 | 5 | medium |
| 32 | Kaliappan, 2013 | 1 | 1 | 1 | 0 | 1 | 1 | 1 | 1 | 7 | medium |
| 33 | Kalra, 1982 | 1 | 1 | 1 | 0 | 1 | 0 | 1 | 1 | 6 | medium |
| 34 | Kearns, 2017 | 1 | 1 | 1 | 1 | 1 | 0 | 1 | 1 | 7 | medium |
| 35 | Lee, 2014 | 1 | 1 | 1 | 0 | 0 | 1 | 1 | 1 | 6 | medium |
| 36 | Lili, 2000 | 1 | 1 | 1 | 0 | 0 | 0 | 1 | 0 | 4 | low |
| 37 | Lyndem, 2002 | 1 | 1 | 1 | 0 | 1 | 0 | 1 | 0 | 5 | medium |
| 38 | Meloni, 1993 | 1 | 1 | 1 | 0 | 0 | 0 | 1 | 0 | 4 | low |
| 39 | Miller, 2018 | 1 | 1 | 1 | 0 | 1 | 0 | 1 | 1 | 6 | medium |
| 40 | Mohd-Shadaruddin, 2018 | 1 | 1 | 1 | 1 | 0 | 1 | 1 | 1 | 7 | medium |
| 41 | Muslim, 2019 | 1 | 1 | 1 | 1 | 1 | 1 | 1 | 1 | 8 | high |
| 42 | Nasr, 2013 | 1 | 1 | 1 | 0 | 1 | 0 | 1 | 1 | 6 | medium |
| 43 | Neo, 1987 | 1 | 1 | 1 | 0 | 1 | 0 | 1 | 0 | 5 | medium |
| 44 | Ng, 2014 | 1 | 1 | 1 | 0 | 1 | 1 | 1 | 1 | 7 | medium |
| 45 | Ngui, 2015 | 1 | 1 | 1 | 1 | 0 | 1 | 1 | 1 | 7 | medium |
| 46 | Ngui, 2016 | 1 | 1 | 1 | 1 | 0 | 0 | 1 | 1 | 6 | medium |
| 47 | Nithikathkul, 2003 | 1 | 1 | 1 | 0 | 0 | 0 | 1 | 0 | 4 | low |
| 48 | Nithikathkul, 2007 | 1 | 1 | 1 | 0 | 1 | 0 | 1 | 0 | 5 | medium |
| 49 | Nor Aini, 2007 | 1 | 1 | 1 | 0 | 1 | 1 | 1 | 1 | 7 | medium |
| 50 | Norhayati, 1995 | 1 | 1 | 1 | 0 | 0 | 0 | 1 | 1 | 5 | medium |
| 51 | Norhayati, 1997 | 1 | 1 | 1 | 0 | 0 | 0 | 1 | 1 | 5 | medium |
| 52 | Norhayati, 1998 | 1 | 1 | 1 | 0 | 0 | 0 | 1 | 1 | 5 | medium |
| 53 | Piangjai, 2003 | 1 | 1 | 1 | 0 | 0 | 1 | 1 | 1 | 6 | medium |
| 54 | Prownebon, 2013 | 1 | 1 | 1 | 0 | 0 | 0 | 1 | 1 | 5 | medium |
| 55 | Rahmah, 1997 | 1 | 1 | 1 | 0 | 1 | 0 | 1 | 0 | 5 | medium |
| 56 | Rajeswari, 1994 | 1 | 1 | 1 | 0 | 1 | 0 | 1 | 0 | 5 | medium |
| 57 | Rajoo, 2017 | 1 | 1 | 1 | 1 | 0 | 1 | 1 | 1 | 7 | medium |
| 58 | Ranjitkar, 2014 | 1 | 1 | 1 | 0 | 0 | 1 | 1 | 1 | 6 | medium |
| 59 | Rao, 2002 | 1 | 1 | 1 | 0 | 0 | 0 | 1 | 1 | 5 | medium |
| 60 | Rao, 2006 | 1 | 1 | 1 | 0 | 1 | 0 | 1 | 1 | 6 | medium |
| 61 | Reynoldson, 1997 | 1 | 1 | 1 | 0 | 1 | 0 | 1 | 1 | 6 | medium |
| 62 | Ribas, 2017 | 1 | 1 | 1 | 0 | 0 | 0 | 1 | 1 | 5 | medium |
| 63 | Ritchie, 1954 | 1 | 1 | 1 | 0 | 0 | 0 | 1 | 0 | 4 | low |
| 64 | Sagin, 2002 | 1 | 1 | 1 | 0 | 0 | 0 | 1 | 0 | 4 | low |
| 65 | Saksirisampant, 2004 | 1 | 1 | 1 | 0 | 1 | 0 | 1 | 1 | 6 | medium |
| 66 | Shield, 2015 | 1 | 1 | 1 | 0 | 0 | 1 | 1 | 1 | 6 | medium |
| 67 | Singh, 1993 | 1 | 1 | 1 | 0 | 1 | 0 | 1 | 0 | 5 | medium |
| 68 | Sinniah, 2012 | 1 | 1 | 1 | 0 | 1 | 0 | 1 | 0 | 5 | medium |
| 69 | Sinniah, 2014 | 1 | 1 | 1 | 0 | 1 | 1 | 1 | 0 | 6 | medium |
| 70 | Stafford, 1980 | 1 | 1 | 1 | 0 | 0 | 1 | 1 | 0 | 5 | medium |
| 71 | Steinmann, 2008 | 1 | 1 | 1 | 0 | 1 | 1 | 1 | 1 | 7 | medium |
| 72 | Sugunan, 1996 | 1 | 1 | 1 | 0 | 0 | 0 | 1 | 1 | 5 | medium |
| 73 | Tienboon, 2007 | 1 | 1 | 1 | 0 | 0 | 0 | 1 | 1 | 5 | medium |
| 74 | Verle, 2003 | 1 | 1 | 1 | 0 | 1 | 0 | 1 | 1 | 6 | medium |
| 75 | Wong, 2016 | 1 | 1 | 1 | 0 | 0 | 0 | 1 | 0 | 4 | low |
| 76 | Yanola, 2018 | 1 | 1 | 1 | 0 | 0 | 1 | 1 | 1 | 6 | medium |
| 77 | Yap, 2012 | 1 | 1 | 1 | 0 | 0 | 1 | 1 | 1 | 6 | medium |
| 78 | Yoshida, 1968 | 1 | 1 | 1 | 0 | 0 | 0 | 1 | 0 | 4 | low |
| 79 | Zulkifli, 1999A | 1 | 1 | 1 | 0 | 1 | 0 | 1 | 1 | 6 | medium |
| 80 | Zulkifli, 1999B | 1 | 1 | 1 | 0 | 1 | 0 | 1 | 0 | 5 | medium |
| 81 | Zulkifli, 2000 | 1 | 1 | 1 | 0 | 1 | 1 | 1 | 1 | 7 | medium |
