# Supplementary material for: The prevalence of soil transmitted helminth infections in minority indigenous populations of South-East Asia and the Western Pacific Region: A systematic review and meta-analysis
Source: PLoS Negl Trop Dis. 2021 Nov 10;15(11):e0009890. doi: 10.1371/journal.pntd.0009890 (PMC8580241; doi:10.1371/journal.pntd.0009890)
Supplement: S3 Table — (DOCX) [file pntd.0009890.s004.docx]

S1 Table D: Key to modified Newcastle-Ottawa Quality Assessment Scale scoring

| Study Population | 0= The study population is not clearly defined |
| --- | --- |
|  | 1=The study population is clearly defined |
| Representativeness of the sample | 0=No description of the sampling strategy. |
|  | 1= Study sample comprises a select group of the study population (non-random sampling) |
|  | 2= Study sample is representative of the study population (all subjects or random sampling) |
| Ascertainment of specimen collection methods | 0= The study does not detail specimen collection methodologies |
|  | 1= The study clearly defines specimen collection methodologies |
| Sample size | 0= Not justified |
|  | 1= Justified and satisfactory (sample size and power calculation included) |
| Non-respondents | 0= No description of the response rate or the characteristics of the responders and the non-responders. |
|  | 1= Comparability between respondents and non-respondents’ characteristics are established. |
| Impact of Bias (selection bias, measurement bias, participant reporting, confounders) | 0= Where appropriate, the study does not acknowledge or mitigate for potential bias. When comparisons are made between different study populations results are not adjusted for confounders |
|  | 1= Where relevant, the study acknowledges and mitigates for potential bias. When comparisons are made between different study populations results are adjusted for confounders |
| Assessment of the outcome (STH infection) | 0= No definitive diagnosis or self-report |
|  | 1= Objective diagnostic methodology with units of measurement and /or definitions |
| Statistical analysis | 0= The statistical test is inappropriate/not described/incomplete |
|  | 1= The statistical method used is clearly described and appropriate for the analysis undertaken. Where comparisons are made between population groups, the measurement of the association is presented, including confidence intervals and the probability level (*p* value) |

The average QA score across STH studies was 5.7 out of a total possible score of 9.
